# Geometric-phase intraocular lenses with multifocality

**DOI:** 10.1038/s41377-022-01016-y

**Published:** 2022-11-02

**Authors:** Seungmin Lee, Gayeon Park, Seonho Kim, Yeonghwa Ryu, Jae Woong Yoon, Ho Sik Hwang, In Seok Song, Chang Sun Lee, Seok Ho Song

**Affiliations:** 1 Department of Physics, Hanyang University, Seoul, 04763 Republic of Korea; 2 Department of Ophthalmology, Catholic University of Korea, Seoul, 07345 Republic of Korea; 3 Seoul Ophthalmic Clinic, Goyang, 10463 Republic of Korea; 4 Koryoeyetech, Inc., Seoul, 06093 Republic of Korea; 5 Tigernics, Inc., Seoul, 04763 Republic of Korea

**Keywords:** Imaging and sensing, Liquid crystals

## Abstract

We demonstrate a new type of multifocal and extended depth of focus (EDOF) intraocular lenses (IOLs) embedding μm-thin geometric phase (GP) lens layers. As an emerging approach for lens phase design, the GP modulated IOLs outperform conventional diffractive IOLs in multifocality while completely avoiding the clinically undesirable demand for additional surface patterns to standard monofocal IOL designs. The number of foci and light splitting ratio of the GP IOLs are adjusted by changing the number of stacked GP layers and the thickness of each layer. Bifocal and trifocal GP IOLs are fabricated by radial alignment of anisotropic orientation in UV-curable liquid crystal polymers. After characterizing the defocus image and modulation transfer function of the GP IOLs, it is expected that GP IOLs will alleviate the most common problems associated with multifocal and EDOF IOLs, blurred vision and photic phenomena caused by light scattering and posterior capsule opacification.

## Abstract (web summary)

Multifocal and extended depth-of-focus intraocular lenses (IOLs) embedding μm-thin geometric phase (GP) layers are demonstrated. GP-IOLs will alleviate blurred vision and photic phenomena of conventional presbyopia-correction IOLs.

## Graphical abstract

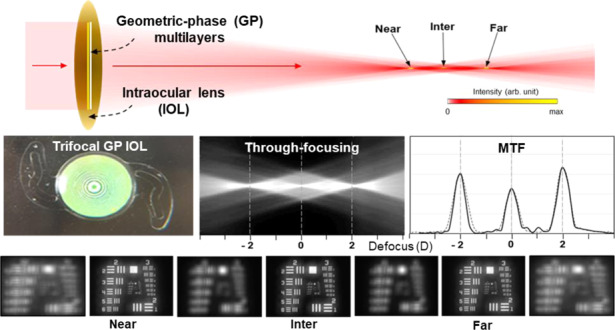

## Introduction

Intraocular lens (IOL) technology for presbyopia correction and cataract surgery is of constantly growing importance as the population aging deepens in many developed societies^1^. An ideal IOL for this purpose is desired to simultaneously support near, intermediate, and distant vision without any serious complications^2,3^. The pseudophakic presbyopia correction presently relies on multifocal or extended depth-of-focus (EDOF) IOLs using combined refractive and diffractive surfaces to create series of axial multi-foci or enhanced field of view^4–6^. In spite of their enormous success, current IOL technologies still have inherent challenging issues for further substantial improvements^7–9^, as described below.

Multifocal intraocular lenses (MF IOLs) work to form multiple focal points by dispersing the energy of light entering the eye, and they have been developed so far by the use of non-physiological optical methods to improve near vision^10^. Most of the industrially accessible MF IOLs are bifocal IOLs with two main focal points for near and far vision, and trifocal IOLs designed by combining two diffraction profiles to improve intermediate vision. In recent years, EDOF IOLs are also getting a good response in the market in an attempt to provide a continuous field of view based on the extension of the distance the eye remains in focus^11,12^. MF and EDOF IOLs can be made of refractive, diffractive or a combination of both designs^13,14^. It is known that diffractive IOL is less dependent on pupil size and more tolerant of kappa angle and decentration, however, their main disadvantage has been the energy lost caused by light scattering at the diffractive surfaces. Diffractive MF IOLs cause approximately 18% of the loss of light in transition and have a high potential of producing halos and glare due to more nontransition areas^7^. These disadvantages may decrease quality of vision, especially in mesopic and scotopic conditions. In particular, the diffusive stray light may lower contrast sensitivity to faint objects and often causes embarrassing halos, glares, and starburst-like optical noises on retina, which are subject to an additional postoperative adaptation period or permanent blurry vision for high spatial-frequency objects^15–17^.

Another issue is the posterior capsule opacification (PCO), which is the most frequent long-term complication of the pseudophakic presbyopia correction^18–20^. PCO is caused by proliferation and migration of lens epithelial cells (LECs) across the posterior capsule and results in severely blurry vision and photic phenomena^21^. It is known that PCO incidence mostly depends on the properties of IOL material, haptic structure, and optic edge angle. However, retrospective analyses to evaluate the correlation of PCO formation with surface roughness of IOLs reveal another interesting claim that the rate of PCO incidence is directly proportional to the increase in surface micro-roughness of IOLs^22,23^. Clinical comparative study also shows that Nd:YAG-laser capsulotomy rate for PCO removal is 3 times higher for multifocal IOL implantation cases than monofocal IOL cases^24,25^. A comparison between two multifocal lenses, diffractive and refractive MF IOLs, reveals that the incidence of Nd:YAG laser capsulotomy in patients with diffractive MF IOLs was higher than that in patients with asymmetric refractive MF IOLs, in addition the diffractive MF OL group increased faster^26^. We note that diffractive MF IOLs essentially involve more complicated, uneven surfaces than refractive MF IOLs, and the uneven surfaces are more likely to cause PCO issues by providing obviously more rooms for inhomogeneous epithelial-cell distributions. Among different diffractive multifocal IOL designs, the capsulotomy rate is remarkably lower for an apodized IOL with smaller average surface-step height. Compare, for example, the rate values 23% for a full-optic diffractive design of AT Lisa tri 839MP (Carl Zeiss Meditec, Jena, Germany) and substantially lower 9% for an apodized diffractive design of FineVision MircoF (PhysIOL, Liège, Belgium), even though these two designs have identical plan-view patterns, i.e., the identical number of discontinuity steps, to produce a common desired refractive-power property^27^. Therefore, it is of great medical and technological interest to develop high-performance multifocal or EDOF IOL structures with minimized surface patterns possibly avoiding the major associated complications^28,29^.

Here, we propose versatile geometric-phase (GP) IOL structures that outperform the conventional diffractive IOLs in the optical functionalities while completely avoiding the clinically undesirable demand for any additional surface patterns to the standard refractive monofocal IOL designs. In our proposed approach, we make use of a flat optical-element technology enabled by the GP effect grounded on the Pancharatnam-Berry phase in polarization-state transformation processes^30–34^. In GP optical elements, desired refractive properties are obtainable by appropriately distributing optically anisotropic domains in a flat μm-thick film. Such GP elements can be easily embedded entirely within conventional refractive IOL structures and, thereby, provide desired additional refractive-power properties in absence of any diffractive surface patterns. A radial, parabolic variation in *ϕ* acts as a lens. Because different handedness of circular polarization makes different signs of the phase shift, beams with one handedness focus and beams of opposite handedness defocus. This GP modulation is physically continuous throughout the spatially variant retardation film due to the unbound nature of GP^35,36^. Thin anisotropic films are continuous down to sub-nanometer scale and can be deposited in multiple layers, ensuring clear, haze-less optics without compromising efficiency and transmittance^37,38^. This is advantageous over the discontinuous surface corrugations of most conventional IOLs. In the proposed MF and EDOF IOLs, GP layers with spatially variant anisotropy axes can be realized using nanostructured metasurfaces^33,39^ or liquid crystal polymers^40,41^. UV-curable liquid crystal polymers (LCP) are particularly attractive because they can be foldable and made at low cost^42,43^.

The basis of GP modulation lies in spatially controlling the local orientation of LCP anisotropic molecules in retardation film^37,44^. Varying the angle *ϕ* of anisotropic orientation makes a 2*ϕ* phase change in circularly polarized incidence^45,46^. Consider a phase retardation due to anisotropy in LCP molecules which is given by *Γ*(*t*, λ) = (2π/λ)(*n*_∥_ − *n*_⊥_)*t*, where λ is wavelength of incident light, and *n*_∥_ and *n*_⊥_ are birefringent index of LCP retardation film with thickness of *t*. When circularly polarized incident light ***J***_±_ = (1, ±*i*) passes through the retardation film whose slow axis is rotated by *ϕ* on the *x*-axis, the Jones matrix **T** and transmitted light ***J***’_±_ with the rotation matrix ***R***(*ϕ*) are given by^46^

1 $$\begin{array}{ll}{{{\boldsymbol{T}}}} &= {{{\boldsymbol{R}}}}( - \phi )\left( {\begin{array}{*{20}{c}} 1 & 0 \\ 0 & {{\rm{e}}^{ - {\rm{i}}\Gamma }} \end{array}} \right){{{\boldsymbol{R}}}}(\phi )\\& = \left( {\begin{array}{*{20}{c}} {{{{\mathrm{cos}}}}\phi } & { - {{{\mathrm{sin}}}}\phi } \\ {{{{\mathrm{sin}}}}\phi } & {{{{\mathrm{cos}}}}\phi } \end{array}} \right)\left( {\begin{array}{*{20}{c}} 1 & 0 \\ 0 & {{\rm{e}}^{ - {\rm{i}}\Gamma }} \end{array}} \right)\left( {\begin{array}{*{20}{c}} {{{{\mathrm{cos}}}}\phi } & {{{{\mathrm{sin}}}}\phi } \\ { - {{{\mathrm{sin}}}}\phi } & {{{{\mathrm{cos}}}}\phi } \end{array}} \right)\end{array}$$

2 $${{{\boldsymbol{J}}}}_ \pm ^\prime = {{{\mathbf{T}}}} \cdot {{{\boldsymbol{J}}}}_ \pm = \frac{1}{2}\left[ {(1 + {\rm{e}}^{ - {\rm{i}}\Gamma })\left( {\begin{array}{*{20}{c}} 1 \\ { \pm i} \end{array}} \right) + (1 - {\rm{e}}^{ - {\rm{i}}\Gamma }){\rm{e}}^{ \pm 2{\rm{i}}\phi }\left( {\begin{array}{*{20}{c}} 1 \\ { \mp i} \end{array}} \right)} \right]$$

The transmitted light ***J***’_±_ acquires additional geometric phase *φ* = 2*ϕ* with an efficiency of converting the polarization handedness given by η(*t*,λ) = sin^2^(*Γ*/2).

We have experimentally developed bifocal and trifocal GP IOLs with a UV-curable liquid-crystal polymer as a foldable anisotropic thin-film material. We experimentally verify that the multifocal GP IOL has higher image visibility and through-focus modulation transfer function (TF-MTF) than a commercial diffractive multifocal IOL. The peak values of TF-MTF for the bifocal and trifocal GP IOLs were found to be approximately 1.4-fold improved compared to commercial diffractive MF IOLs^16^, revealing the advantages of GP IOLs with significantly lower image blur and scattering loss. Importantly, the proposed GP IOL structure allows multiple GP stacks for enhanced number of foci at arbitrary desired locations and multifocal EDOF diversification without any significant optical power loss as opposed to the diffractive surface-pattern approaches. The conversion efficiency of incident light energy to the bifocal planes can still be 97.6% even if the layer thickness suffers a 10% deviation^46^. Therefore, we expect that the GP MF and EDOF IOLs can settle the most common problems associated with multifocal lenses, blurred vision, and photic phenomena, by reducing light scattering and PCO.

### Multifocality of GP IOLs

**Fig. 1:**
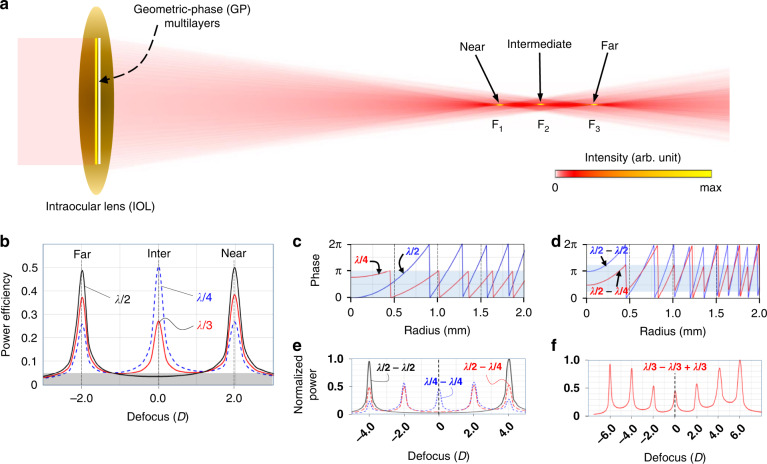
Design of GP IOLs. **a** Schematic of a GP IOL and exemplary trifocal generation from the field-tracing numerical analysis. When a single GP layer is embedded, the two states of circular polarizations (CP), right-handed CP and left-handed CP, of an unpolarized light incident to the GP IOL are focused on either one of F_1_ and F_3_ focal planes, respectively, while the mixed CPs on the middle plane of F_2_. **b** Through-focus power efficiency curves along the optical axis for three different trifocal GP IOLs with relative OPR Δ*L*_1_ = λ/2, λ/3, and λ/4, respectively. The λ/2 OPR design has two even-intensity foci at Far and Near defocusing-power positions while the λ/3 and λ/4 OPR designs have three foci at Far, Inter, and Near positions with different intensity allocations. **c** Radial phase-retardation profiles of GP IOLs for λ/2 and λ/4 OPR designs. **d** Radial phase-retardation profiles of bilayer GP designs for OPR combinations (Δ*L*_1_, Δ*L*_2_) = (λ/2, −λ/2) and (λ/2, −λ/4), where “−” represents the phase profile flipped to negative slope. **e**, **f** Through-focus power distribution curves of three bilayer- GP IOLs for (Δ*L*_1_, Δ*L*_2_) = (λ/2, −λ/4), (λ/4, −λ/4), and (λ/2, −λ/2), and one trilayer-GP IOL with (Δ*L*_1_, Δ*L*_2_, Δ*L*_3_) = (λ/3, −λ/3, λ/3). The power is normalized to the maximum of the peak power

The proposed GP IOL structure consists of a refractive monofocal IOL as a high refractive-power base lens and embedded GP layers providing additional low-power refraction for focus multiplication or depth-of-focus diversification (Fig. 1a). Therein, we include a numerically calculated optical intensity distribution for unpolarized incident light as an exemplary case of a single birefringent GP-layer inclusion that produces three evenly distributed foci in the absence of any additional surface patterns. The unpolarized incident light used in the calculation is represented by a combination of two states of circular polarizations (CP), right-handed CP and left-handed CP. Each of the GP layers acts as a lens composed of an anisotropic thin film with a radial, parabolic variation in anisotropic orientation angle *ϕ*. The two states of right-handed and left-handed CPs incident to the GP IOL are focused on either one of F_1_ and F_3_ focal planes, respectively, while the mixed CPs on the middle plane of F_2_^46^. Details in *ϕ* distribution and the relationship between handedness CP and foci will be described later (Fig. 4). The GP IOL design and the numerical analysis presented in this paper were performed using a field-tracing method^47–49^ (“Methods”).

For birefringent GP layers, relative optical path retardation (OPR) Δ*L*_*j*_ = Δ*n*·*t*_*j*_ is a key parameter that controls optical power distribution over generated multi foci, where Δ*n* = *n*_∥_ − *n*_⊥_ is the principal refractive-index difference and *t*_*j*_ is thickness of the *j*th layer as described in Eq. (2). For monolayer GP-IOLs with different OPRs at Δ*L*_1_ = λ/2, λ/3, and λ/4, the through-focus power efficiency, defined as the measured power at each defocus point normalized to the incident power, clearly shows bifocal and trifocal peaks on the discrete defocusing powers (D) (Fig. 1b). The sum of power efficiencies at the three foci (Far, Inter, Near) is nearly unity (100%) for all three OPRs, indicating that multifocal GP IOLs can produce negligible scattering losses. In further detail, refractive power addition due to a GP element in this design depends on the handedness of circular polarization (CP) component such that added refractive powers (add power hereafter for convenience) for one CP handedness and the other take an identical magnitude but opposite signs, respectively. Therefore, a certain portion of one CP handedness in a reference focus (Intermediate focus, F_2_) from the base monofocal refractive IOL is transferred to an additional focus with a higher refractive power (Near focus, F_1_) and another certain portion of the other CP handedness is transferred to another additional focus with a lower refractive power (Far focus, F_3_), while the mixed CP to the mid-plane of F_2_ (Inter) if Δ*L*_1_ deviates from λ/2^50^. The amount of the transferred CP component from the reference focus to an additional focus depends on Δ*L*_*j*_ such that it reaches the maximum at 100% for Δ*L*_*j*_ = (*m* + 1/2)λ or the minimum at 0% for Δ*L*_*j*_ = *m*λ, where *m* is an integer. Therefore, one can conveniently obtain an arbitrarily desired focal-power distribution by appropriately tuning Δ*L*_*j*_ value between 0 and λ/2 (Fig. 1b).

The phase profiles of the λ/4 and λ/2 GP layers, which provide the −2.0 D add powers for left-handed CP, consist of diffractive concentric zones getting closer away from the center (Fig. 1c). The +2.0 D add powers for right-handed CP are negative and composed by the phase profiles flipped to negative slope. The λ/4 and λ/2 GP layers can be stacked together to form more complex phase profiles (Fig. 1d), where “−” represents the inverted phase profile. Remarkably, multiple stacks of these GP layers produce a very interesting distribution of multifocals (Fig. 1e, f), for example, 2 or 3 foci in a double stack and 7 foci in a triple stack.

**Fig. 2:**
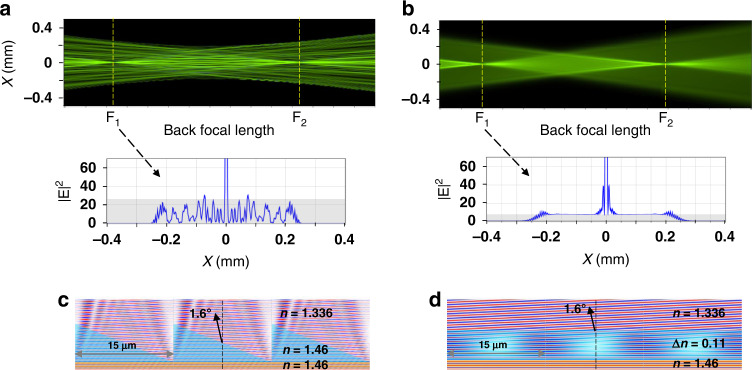
Comparison of light scattering from conventional diffractive IOL and GP IOL for a coherent beam incidence. **a**, **b** Through-focus point spread functions of diffractive and GP bifocal IOLs, respectively. The defocus distance between F_1_ and F_2_ is 4 D. The point spread functions on F_1_ focal plane along the *x*-axis are compared bellow. **c**, **d** Propagation of planewave normally incident from the bottom through a diffractive sawtooth profile and uniformly thick GP film (blue areas), respectively. After passing through the phase modulation layer, the GP film produces very uniform wavefronts while the incident beam is deflected at a certain angle of 1.6° in both cases

Comparing the scattered light of a conventional diffractive bifocal IOL^7^ and the λ/2 OPR GP IOL (Fig. 2a, b), the background-to-peak power ratio of the GP IOL is 2.4% and the background level (gray area) is very flat. On the other hand, the ratio of the diffractive IOL is about 7.2%, which is very noisy. This intense noise around the foci mainly come from light scattering created by the discontinuous-step blazed profile of the conventional IOL, whereas the GP film with the same blazed phase profile for a circularly polarized light produces very uniform wavefront (Fig. 2c, d). As the blazed height continues to increase from 0 to 2λ (4π), the scatted noise also increases, whereas as the thickness of the GP film increases the wavefront of the transmitted field remains very uniform (Supplementary Movie 1). These flat and uniform wavefronts after transmission are originated from the unbounded nature of GP gratings^36^, as it allows for continuous incremental modulation of the geometric phase up to arbitrary magnitudes, not restricted to 2π.

### Fabrication of GP IOLs

**Fig. 3:**
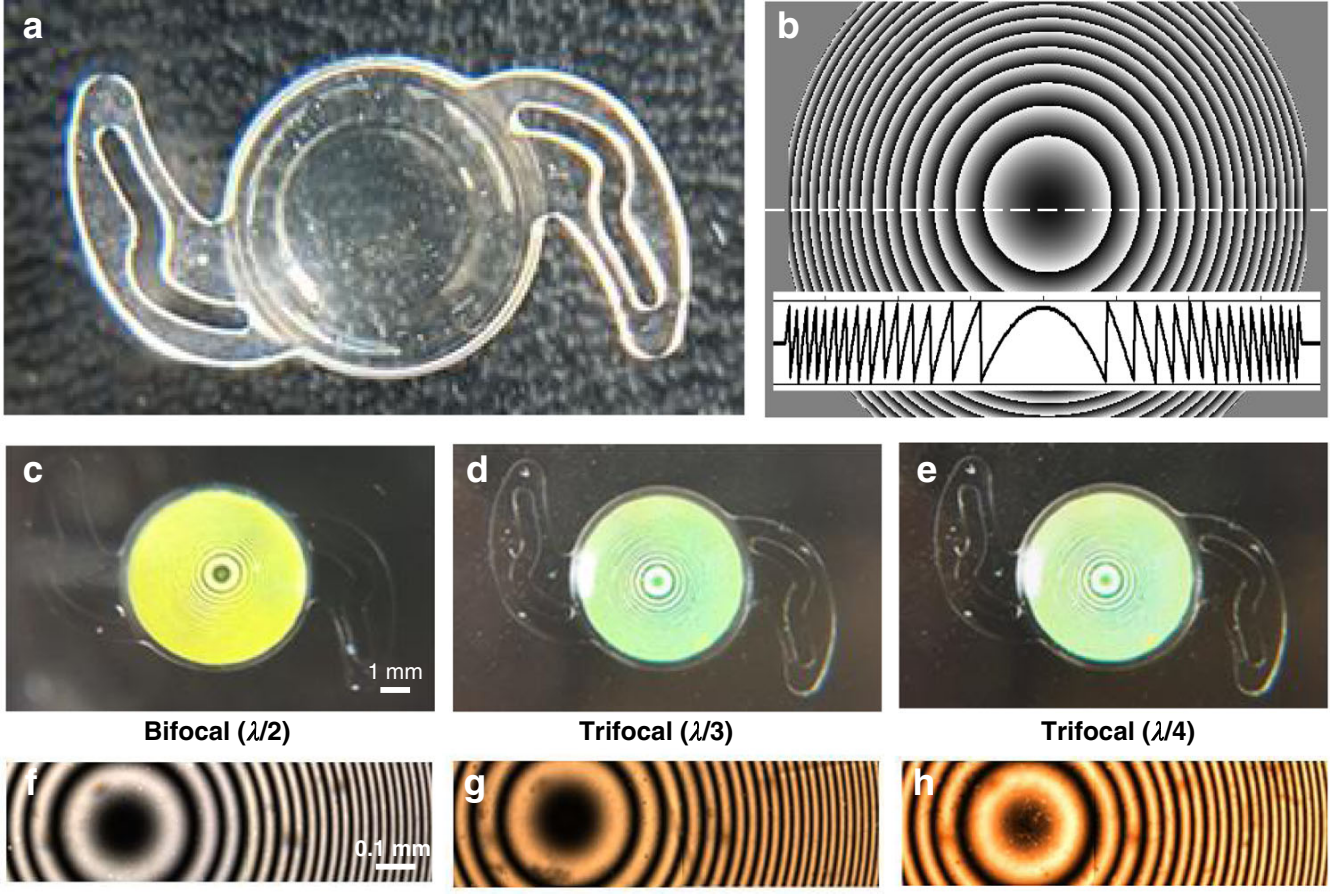
Fabrication results of bifocal and trifocal GP IOLs. **a** Photograph of the hydrated GP IOL. Overall diameter is 12.0 mm and optic diameter is 6.0 mm. Base power is 20.0 D. **b** Designed phase distribution of the GP layer with 2.0 D add power. The inset shows the phase profile along the central cross section (dashed horizontal line). Measured polarizing optical micrographs under crossed polarizers, where bifocal (λ/2 OPR) in (**c**, **f**), trifocal (λ/3 OPR) in (**d**, **g**), and trifocal (λ/4 OPR) in (**e**, **h**)

A bifocal GP IOL with λ/2 OPR and two trifocal GP IOLs with λ/3 and λ/4 OPRs were fabricated (“Methods”). The hydrophilic acrylic base lens with optic diameter (6.0 mm) and center thickness (0.72 mm) has a base power of 20 D, and the overall length of IOLs including the two haptic parts is 12 mm (Fig. 3a). The GP layer embedded IOLs appears transparent but has a full phase modulation from 0 to 2π (Fig. 3b). The phase modulation can be visualized by the polarizing optical micrographs measured under crossed polarizers, where the different colors represent the deference in OPR (Fig. 3c–e). The magnified fringes of the concentric rings are chirped radially and the brightness gradient represents the phase change of transmitted light from 0 to 2π (Fig. 3f–h). The same chirped profiles mean that all GP layers of the multifocal IOLs provide a same add power of +2.0 D or −2.0 D depending on the handiness of incident circular polarization.

**Fig. 4:**
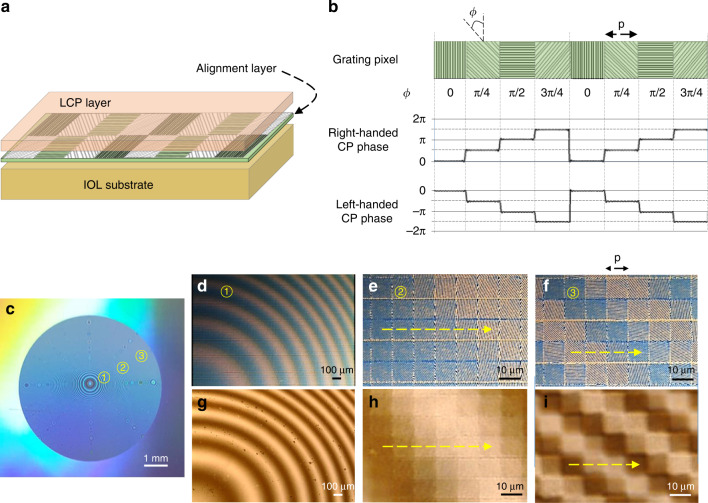
Fabrication of GP layers. **a** Birefringent LCP layer spin-coated onto alignment layer of nanopatterned surface imprinted on IOL substrate. **b** The nanopatterned surface composed of square grating pixels with discrete corrugation angles (*ϕ*) and constant width (*p* = 10 μm), and the corresponding GP modulation (2*ϕ*) for right-handed CP and left-handed CP incidences. **c** Photograph of the microtextured grating pixels fabricated on a silicon wafer. **d**–**f** Photographs of the square grating pixels at the positions of ①, ②, and ③ marked in (**c**), respectively. **g**–**i** Measured polarizing optical micrographs of the aligned LCP layer under crossed polarizers at the regions respective to (**d**–**f**). The brightness represents the geometric-phase distribution normalized by 2π

Spatial control of the local orientation of anisotropic molecules in the GP layer was performed by a nanopatterned surface using nanoimprinting lithography (Supplementary Fig. S1). Birefringent LCP material (reactive mesogen, RMS03-013, Merck) is spin-coated onto the alignment layer of nanopatterned surface imprinted on IOL substrate (Fig. 4a). The nanopatterned surface is composed of square grating pixels (grating period of 500 nm) with discrete corrugation angles (*ϕ*) and constant width (*p* = 10 μm) (Fig. 4b). As *ϕ* rotates stepwise along the grating pixels, the LCP coated on the alignment layer changes from inhomogeneous in plane, copying the *ϕ* directions, to a discrete configuration with a full alignment range of *ϕ* = 0°–180° and an angle resolution of Δ*ϕ* = 0.044° (Supplementary Table S1). The right-handed CP component of light after transmitting the aligned LCP layer acquires phase shift of 0–2*ϕ*, while the left-handed CP does opposite. The nanopatterned alignment layer with 6 mm in diameter and 150 nm in grating depth is imprinted on an IOL button (disc-type raw material, CI26, Contamac Ltd.) (Fig. 4c). Magnified microscope images of the grating pixel distribution at the positions of ①, ②, and ③ in the IOL button (Fig. 4d–f) show the variation in brightness, which reveals the distribution of *ϕ*, for example, *ϕ* = 45°–0° (along the yellow dashed line in Fig. 4e) and *ϕ* = −45°–45° (Fig. 4f). After the birefringent LCP layer is spin-coated onto the alignment layer of nanopatterned surface, the GP modulations of 2*ϕ*’s at the three marked positions can be confirmed by the polarizing optical micrographs (Fig. 4g–i).

### Through-focus efficiency and modulation transfer function of GP IOLs

**Fig. 5:**
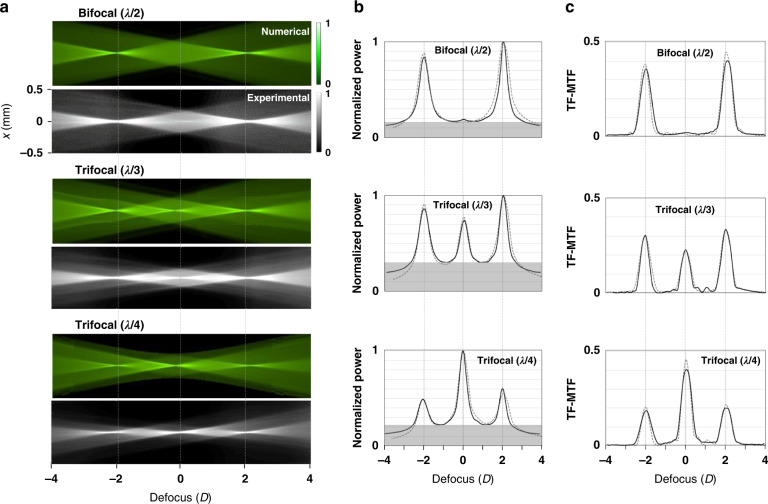
Measurement results of focal efficiency and TF-MTF. **a** TF-PSF of the 3 GP IOLs evaluated on a 1.0 mm detection window within a defocus range of −4 D to +4 D from a base power of 20 D. The numerical (green) and experimental (gray) TF-PSFs show good agreement with each other even at the focus periphery. **b** Normalized power distributions experimentally measured along the propagation axis at *x* = 0 mm. The shaded area at the bottom means a background due to intrinsic halo effect of multifocal IOLs. These PEs also correlate well with the numerical ones in Fig. 1c. **c** TF-MTF experimentally measured at 50 line pairs/mm frequency with a 25 μm slit object. For comparison, the normalized power and TF-MTF (dotted curves) numerically calculated by a field tracing method are also presented in (**b**, **c**)

The optical performance of the three fabricated GP IOLs at a pupil diameter of 4 mm was experimentally tested in vitro with an optical bench setup^51^ (Supplementary Fig. S2). The through-focus point spread functions (TF-PSF) measured in the experiment showed good agreement with the numerical ones (Fig. 5a, b). There was almost negligible random scattered light in all defocus ranges thanks to the smooth lens interfaces and flat GP layers. Uniform background light levels (gray areas in Fig. 5b, approximately 16% for peaks at bifocal and 20–30% at trifocals) observed in well-balanced multifocals after measuring the through-focus normalized power along the propagation axis. A 25 μm wide cross slit is used as a target to obtain their TF-MTFs at 50 line pairs/mm (Fig. 5c). For comparison, the MTF corresponding to a monofocal IOL with +20 D base power is considered as a reference (not shown here). The peak values of the TF-MTF at the 3 focal planes (−2.0 D, 0 D, +2.0 D) remain reasonably within a range of 0.2 to 0.4. The sum of two peak values of the bifocal MTF at −2 D and +2 D is 0.76 and that of three peaks of the trifocal (λ/3) at −2 D, 0 D, and +2 D is 0.86. Those peak values of TF-MTF for the bifocal and trifocal GP IOLs are approximately 1.4-fold improved compared to commercial diffractive MF IOLs^16^, demonstrating the advantages of GP IOLs with significantly lower image blur and scattering loss.

### Through-focus imaging of GP IOLs

**Fig. 6:**
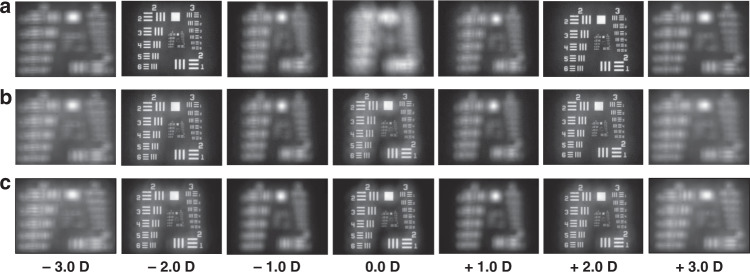
Defocus imaging of the GP IOLs. Through-focus images of a US Air Force (USAF) target measured in 1.0 D steps at 4 mm pupil aperture. **a** The two images at −2.0 D and +2.0 D measured with the bifocal GP IOL are clearly visible. **b**, **c** Trifocal images with the λ/3 and λ/4 GP IOLs, respectively. Those at 0.0 D correspond to intermediate visions

Through-focus images of the US Air Force (USAF) target were measured by using the bifocal and trifocal GP IOLs (Fig. 6a–c). At 4 mm pupil diameter, two best images were obtained at −2.0 D and +2.0 D for the bifocal (λ/2) and another one at 0.0 D for the two trifocals (λ/3 and λ/4). The image contrast is proportional to the magnitude of the TF-MTF values (Fig. 5c). For example, for the trifocal (λ/3), the two TF-MTF peak values of about 0.3 at D = −2 and +2 show higher contrast in imaging than the TF-MTF value of 0.22 at *D* = 0. This image contrast is inverted in the trifocal (λ /4) GP-IOL. Although the defocus changes continuously, the images have the same size thanks to the Badal lens in the optical bench setup (Supplementary Movie 2). The visibility, defined as the ratio of (*I*_max_ − *I*_min_)/(*I*_max_+ *I*_min_) where *I*_max_ and *I*_min_ are the maximum and minimum intensities at the 3 bars (Group #4, Element 1 in the target), was 0.51 at far vision (−2.0 D) and 0.48 at near (+2.0 D) for the bifocal IOL (Supplementary Fig. S3a). The 3-bar target pattern almost corresponds to 50 lines/mm spatial frequency for the optical setup. The far and near visibilities for the λ/3 and λ/4 GP IOLs IOL (Supplementary Fig. S3b, c) were (0.49, 0.41) and (0.39, 0.40), respectively, which are slightly lower than the bifocal case due to additional intermediate imaging at 0 D. Comparing the intermediate visibility between the two trifocals, the λ/3 and λ/4 GP IOLs have 0.39 and 0.46, which also tends to match the TF-MTF results. The main reason the absolute values were measured differently could be due to aliasing associated with a discrete sampling.

### Double-layered GP IOLs

**Fig. 7:**
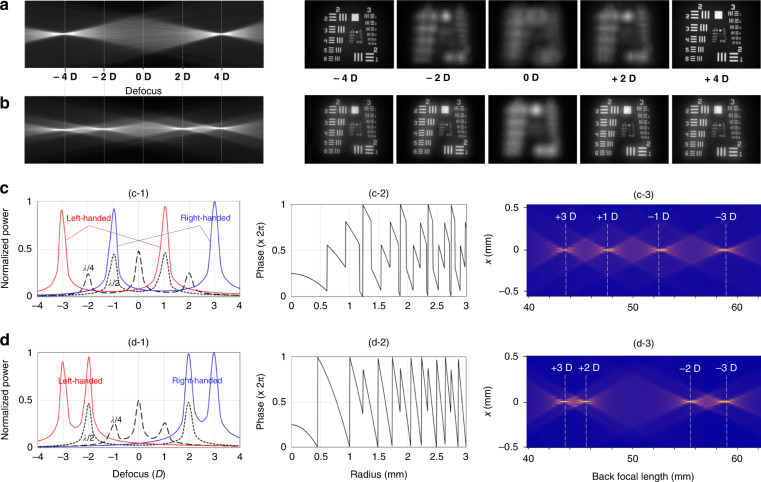
Double-layered GP IOLs. **a**, **b** Double-layered GP combinations of (Δ*L*_1_, Δ*L*_2_) = (λ/2, −λ/2) and (λ/2, −λ/4) with a same add power of 2.0 D, respectively. TF-PSF (left) and defocus images (right) of (λ/2, −λ/2) GP combination in **a** show two foci and two clear images measured at −4 D and +4 D, while those of (λ/2, −λ/4) GP combination in **b** show four foci and four clear images at −4 D, −2 D, +2 D, and +4 D. **c**, **d** Double-layered GP combinations of (Δ*L*_1_, Δ*L*_2_) = (λ/4, −λ/2) and (λ/2, −λ/4) with different add powers, respectively, where the positive OPR layers of λ/4 and λ/2 have a 2.0 D add power and the negative OPR layers of −λ/2 and −λ/4 have a 1.0 D add power. **c**-**1**, **d**-**1** Normalized power distributions of the (λ/4, −λ/2) and (λ/2, −λ/4) combinations, respectively. For comparison, power distributions of single-layered GP cases of λ/4 (2.0 D) and λ/2 (1.0 D) are presented by the dashed and dotted curves, respectively. **c**-**2**, **d**-**2** Combined phases of the (λ/4, −λ/2) and (λ/2, −λ/4) combinations, respectively. **c**-**3**, **d**-**3** TF-PSFs for unpolarized light incidence

Stacking multiple GP layers with different thicknesses can create interesting multifocal distributions. For example, two double-layered GP combinations of (Δ*L*_1_, Δ*L*_2_) = (λ/2, −λ/2) and (λ/2, −λ/4) with a same add power of 2.0 D reveal the radial phase-retardation profiles (Fig. 1d) and the distinct multiple foci (Fig. 1e). We fabricated the two double-layered GP IOLs and measured their TF-PSFs and defocus images (Fig. 7a, b). The (λ/2, −λ/2) combination produces two clear images at −4 D and +4 D, and the 8 D defocus gap between the two foci is twice as wide as the single-layer case (Fig. 1b). The (λ/2, −λ/4) combination, on the other hand, acts as a quadrifocal IOL generating four images at −4 D, −2 D, +2 D, and +4 D.

We can further expand the double-layered concept of GP IOLs for quadrifocal (Fig. 7c, d). A balanced peak power and equal defocus spacing between four foci can be achieved (Fig. 7c), where the two layers provide different add powers: the first λ/4 GP layer has a 2.0 D add power (dashed curve in Fig. 7c-1) and the second λ/2 GP layer has a 1.0 D add power (dotted curve). The combination of the (λ/4, −λ/2) GP layers creates a four-stepwise phase profile (Fig. 7c-2), resulting in four balanced foci at +3 D, +1 D, −1 D, and −3 D from a base power of 20 D for unpolarized light incidence (Figs. 7c-3). Since different handedness of circular polarization makes different signs of the phase shift, it focuses a beam of one handedness at −3 D and +1 D foci while the beam of opposite handedness at −1 D and +3 D (Fig. 7c-1). On the other hand, if the two GP layers are stacked in the order of λ/2 (2.0 D)−λ/4 (1.0 D), the composited phase (Fig. 7d-2) is very different form the previous one. The spike-wise phase creates similar four balanced foci, but with a wide gap in the middle (Fig. 7d-1, d-3).

### EDOF GP IOLs

**Fig. 8:**
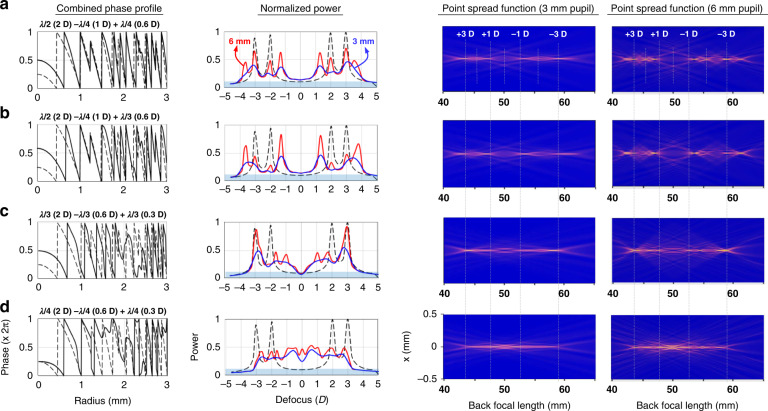
EDoF GP IOLs. Triple-layered GP IOLs stacked in order of λ/2 (2 D)−λ/4 (1 D)+λ/4 (0.6 D) (**a**), λ/2 (2 D)−λ/4 (1 D)+λ/3 (0.6 D) (**b**), λ/3 (2 D)−λ/3 (0.6 D)+λ/3 (0.3 D) (**c**), and λ/4 (2 D)−λ/4 (0.6 D)+λ/4 (0.3 D) (**d**), where the ‘*x* (*y*)’ notation represents the OPR (add power) of the stacked GP layers. The dashed black curves in the combined phase profile and the normalized power diagrams are those for the double-layered GP IOL of λ/2 (2 D)−λ/4 (1 D) (Fig. 7d) for comparison. The red and blue curves of normalized power correspond to the use of pupil diameters of 6 and 3 mm, respectively, and the blue lower area is the background caused by the halo effect. The point spread functions are displayed on equal scales of 0 to 4 color brightness, and the 50 mm back focal length of the IOLs means 0.0 D position

Triple-layered GP IOLs can create EDOF behavior (Fig. 8a–d). First consider two different combinations of triple GP layers: one is in order of λ/2 (2 D)−λ/4 (1 D)+λ/4 (0.6 D) (Fig. 8a), and the other is λ/2 (2 D)−λ/4 (1 D)+λ/3 (0.6 D) (Fig. 8b), where the ‘*x* (*y*)’ notation represents the OPR (add power) of the stacked GP layers. The combined phase profiles (left column) are similar but slightly different at radii > 2 mm, so the through-focus normalized power (middle column) and point spread function (two right columns) for a pupil diameter of 3 mm are hardly distinguishable indistinguishable in the −5 D to +5 D defocus range. Two groups of 4 peaks in opposite defocus ranges, with a wide central gap, merge together as the pupil diameter decreases, extending the depth of focus.

The situation is more dramatic when another two triple-layered GP IOLs of λ/3 (2 D)−λ/3 (0.6 D)+λ/3 (0.3 D) (Fig. 8c) and λ/4 (2 D)−λ/4 (0.6 D)+λ/4 (0.3 D) (Fig. 8d) are compared. In the mid-radius range, the aperiodic freeform-shaped phase profile further extends the EDOF region, and in the last case appears almost continuous over the entire defocus range with a power contrast of more than 0.8. The point spread function for the 3 mm pupil shows an undiffracted Bessel beam with a very long depth-of-focus length from +3 D to −3 D, corresponding to the full range of vision possible with a multifocal IOL. It is worth noting that the combined phase profile containing a freeform located in the paracentral part changes the wavefront of the central light beams to elongate the depth of focus. This wavefront shaping effort is similar to the X-WAVE technology (AcrySof IQ Vivity, Alcon Laboratories, Inc.) that uses two non-diffractive transition elements with smooth elevation changes in the 1 µm range, located in the paracentral portion of optical zone (AcrySof® IQ VivityTM Extended Vision IOL DFU). Our results show that a wider freeform paracentral segment works synergistically and simultaneously to create a longer, continuous extended focal range.

## Discussion

A new type of multifocal IOL is implemented based on the geometric phase (GP) concept, also known as Pancharatnam-Berry phase, which is an emerging approach for designing the phase modulation profile of optical lenses. We demonstrated bifocal and trifocal GP IOLs embedding an extra μm-thin GP modulation film while retaining the smooth anterior and posterior surfaces of the IOL profiles. The GP film with spatially variant anisotropy axes were realized using nanostructured surfaces for aligning a reactive mesogen of UV-curable liquid crystal polymers (LCP). GP IOLs have an advantage over most conventional IOLs with discontinuous surface corrugations as LCP films are continuous down to the sub-nanometer scale and can be deposited in multiple layers, ensuring transparent and haze-less optics without compromising efficiency and transmittance. By measuring the TF-MTF and imaging contrast of the mutifocal GP IOLs we confirmed that the multifocality can be improved by about 1.4-fold compared to the commercial diffractive multifocal IOLs, indicating the advantage of significantly lower image blur and scattering loss of the GP IOLs.

Stacking multiple GP modulation layers with different thicknesses and alternating phase gradients can also create more interesting multifocality. Double-layered GP IOLs can be quadrifocal with balanced power efficiency, and tripled-layered ones can make an extension of focal depth. The combined GP phases are still periodic in a square radius like a lens, but have free-form envelops in the middle. Thus, combining multiple GP layers allows to control multifocality of IOLs with arbitrary topological profiles that may be very difficult to achieve in conventional multifocal IOLs. The idea of stacking multiple discrete LCP layers with certain angles between their optical axes or opposite slopes between their material dispersions can be applied to broaden the bandwidth of GP IOLs over visible wavelengths^52^. The efficiency for even the simplest single-layer GP grating can be greater than 95% in more than half of the 400–700 nm band of visible wavelengths. Another chromatic aberration of GP IOLs is caused by the angular dispersion after passing through the GP layer. The exit propagation direction is related to the gradient of the GP profile. For a GP lens with focal length of *f*_0_ designed for wavelength λ_0_, parallel light can still be focused with a modified focal length, *f* (λ) ~ *f*_0_ (1 − Δλ/λ_0_), for Δλ/λ_0_ ≪ 1. This means that GP IOLs are always chromatic, as in conventional diffractive IOLs, even though their efficiency can be made broadband by using multiple layers.

For the alignment layer on GP IOL substrate, the individual building blocks of square grating pixels were 10 μm in size. The optical efficiency may be affected by this phase discretization, particularly for low *f*-number lenses. Higher resolution patterning for alignment should be advantageous, leading to ideal optical efficiency. However, since the GP layer embedded in the multifocal GP IOLs has a typical *f*-number of about 50 for generating 4 D additional power, the efficiency degradation due to the 10 μm phase discretization is less than 2%^46^. Also note that, due to the focusing efficiency characteristics of GP IOLs that depend on the circular polarization state of incident light, some mobile phones or tablet PCs may cause problems. For example, the iPhone6 display panel (Apple, Cupertino, CA) usually emits right circularly polarized light while the Galaxy Z Flip (Samsung, Suwon, South Korea) emits linearly polarized light. Therefore, near vision may be better for iPhone6, otherwise special attention may be paid to the display polarization state, such as by attaching a transparent waveplate film to the display glass^9^.

Biocompatibility of the LCP material (RMS03-013C Licrivue) used in the GP layers and the human eye has not yet been established clinically. However, it is promising to note that test results of the LCP material reported in the Material Safety Data Sheet (SDS No. 70MDGM136709, Merck) show negative genotoxicity in vivo and no irritation to rabbit skin and eyes. The fact that the LCP layer of a multifocal GP IOL can be inserted between the lens materials also helps to improve long-term capsular biocompatibility. If the radius of the LCP layers is smaller than the basic lens, the upper and lower buttons of the transparent HEMA are copolymerized so that they are not exposed to the outside after lathing the composite button. Another consideration that may affect the GP IOL performance is the corneal polarization of the living human eye, which is described as biaxial anisotropy^53^. The ocular structure is likely to cause a change in the polarization state. The corneal stroma is made up of about 50 to 100 layers of parallel fibers, each of which is known to be birefringent. A preferential orientation exists because the fiber layers may not be completely oriented at random. Since the difference in biaxial refractive index along the 3 axial directions is only about 0.01^54^, the corneal anisotropy may not produce a meaningful effect on the GP IOL multifocality.

In conclusion, the proposed GP-IOL structure allows multiple GP-element stacks for enhanced number of foci at arbitrary desired locations and multifocal EDoF diversification without any significant optical power loss as opposed to the diffractive surface-pattern approaches. We expect GP multifocal and EDOF IOLs to reduce light scattering and possible PCO, thereby addressing the most common problems associated with multifocal lenses, blurred vision, and optical phenomena.

## Methods

### GP IOL design

We implemented the field tracing operators in the physical optics simulation and design software VirtualLab Fusion^49^, and all simulations for GP IOLs are performed in this software. The incident light is monochromatic with a wavelength of 546 nm and a beam diameter of 6 mm (Fig. 1a). The lens material is a hydrophilic acrylic base with a refractive index (*n*) of 1.46, placed in an ambient medium (*n* = 1.336). The anterior (front) surface of the base lens has a radius of curvature of 10.742 mm and a diameter of 6 mm, and the posterior (rear) has a radius of −14.550 mm, a cone constant of −1.0228, a diameter of 6 mm, and the center thickness of the lens is 0.717 mm. The base power of the lens is 20 D, which means that the back focal length (F_2_ focal plane in Fig. 1b) is 66.8 mm. The back focal lengths for −2 D defocus (F_1_ focal plane) and +2 D defocus (F_3_ focal plane) are 60.7 and 74.2 mm, respectively. The difference in the anisotropy index (Δ*n* = *n*_∥_ − *n*_⊥_) of the GP medium is 0.11 (*n*_∥_ = 1.69, *n*_⊥_ = 1.58), so the physical thicknesses of the λ/2, λ/3, and λ/4 OPRs are 2.48, 1.65, and 1.24 μm, respectively. The GP layers with 6 mm diameter made out LCP media with spatially varying rotation of anisotropic axial rotation with an angular resolution of 0.044° were represented by using a programming interface in the software.

### GP IOL fabrication

The core technology for manufacturing LCP based GP lens depends on how well the alignment axis direction of LCP birefringent molecules can be spatially arranged. Photoalignment method with polarization holography or by rastering a laser beam while simultaneously controlling the polarization orientation has been widely used for high-quality and high-resolution multidomain orientation of LCP molecules^44^. As an alternative, LCP molecules can also be aligned by employing specific surface topographies, which can be created by microscale or nanoscale patterns, allowing more freedom in the control of alignment properties and mass production^55,56^.

Here we adopted laser interference lithography (LIL) and nanoimprinting techniques to fabricate nanoscale patterns with arrays of 500 nm period grating pixels^57,58^. To generate silicon molds for nanoimprinting, we developed a home-built LIL system (Supplementary Fig. S1a), consisting of a UV (351 nm) pulsed laser (Nd:YLF AONano 351-3-2-CY, Advanced Optowave Co.), a pair of acousto-optic deflectors (AOD, DTSX-400–405, AA Opto-Electronic Co.), and an XY motorized stage (450 mm × 450 mm, INNO6 Co.). By adjusting the interference angles of *θ* and *ϕ* with AODs, period (Λ) and orientation (*ϕ*) of nanoscale gratings can be precisely defined within the ranges of *Λ* = 0.3 to 3 μm and *ϕ* = 0 to 180° (Supplementary Fig. S1b and Table S1). A typical micrograph of squared grating pixels made of photoresist (AZ5206, MicroChemicals) on a silicon wafer shows a grating array of 100 nm-deep parallel corrugations, which have various pairs of Λ and ϕ but are very uniformly formed within every ~10 μm^2^ pixel area (Supplementary Fig. S1c).

The silicon mold for manufacturing GP lens with 6 mm diameter was fabricated by using the LIL system (Fig. 4c–f). The grating period is fixed at 500 nm for all 10 μm^2^ grating pixels, while the grating orientation angles ϕ are spatially varying in radial. A polymer film replica of the silicon mold is then used as a template for nanoimprinting the grating pixels on a hydrophilic acrylic IOL substrate (IOL button) with 16 mm in diameter and 3 mm in thickness (CI26, Contamac Ltd.). A mixture (RMS03-013, Merck) containing an initiator, reactive liquid-crystalline monomer, and a volatile organic solvent is spin-coated on an alignment layer nanoimprinted on the IOL substrate. Upon drying, the reactive mesogens are then polymerized to be an aligned LCP layer by exposing the sample to radiation that initiates the polymerization process^59^ (Fig. 4a). These thin film coatings are continuous to sub-nanometer scale ensuring clear, haze-less, and multilayer GP lenses without compromising efficiency and transmission. Notably, the nature of LC anchoring in the pixelated grating surface is different from that of the more commonly encountered homogeneous surfaces on which the axis of symmetry is constant everywhere^55^. The LC director residing within the grooved alignment layer is forced to deform according to the checkerboard grating pixels. Such a picture of the surface potential gains enough support for the in-plane uniform alignment within the pixel boundary (Fig. 4g–i). Finally, after attaching another blank button on the LCP layer aligned on the IOL button, we cut it into a lens shape by lathe operation (Optoform 80, Ametek Precitech, Inc,).

## Supplementary information

Supplementary Information

Movie 1

Movie 2

## Data Availability

The main data supporting the results of this study are available within the paper and its Supplementary Information. Other raw data generated during this study are available from the corresponding author on reasonable request.
