# Supplementary material for: Geometric-phase intraocular lenses with multifocality
Source: Light Sci Appl. 2022 Nov 2;11:320. doi: 10.1038/s41377-022-01016-y (PMC9630405; doi:10.1038/s41377-022-01016-y)
Supplement: Supplementary file 1 — Supplementary Information [file 41377_2022_1016_MOESM1_ESM.docx]

**(Supplementary Information)**

**Geometric-phase intraocular lenses with multifocality**

Seungmin Lee^1^, Gayeon Park^1^, Sunho Kim^1^, Yeonghwa Ryu^1^, Jae Woong Yoon^1^,

Ho Sik Hwang^2^, In Seok Song^3^, Chang Sun Lee^4^ and Seok Ho Song^1,5 *^

^1^Department of Physics, Hanyang University, Seoul 04763, Republic of Korea

^2^Department of Ophthalmology, Catholic University of Korea, Seoul 07345, Republic of Korea

^3^Seoul Ophthalmic Clinic, Goyang 10463, Republic of Korea

^4^Koryoeyetech, Inc., Seoul 06093, Republic of Korea

^5^Tigernics, Inc., Seoul 04763, Republic of Korea

* e-mail: shsong@hanyang.ac.kr

(Contents)

Figure S1. Laser interference lithography system.

Figure S2. Badal imaging system.

Figure S3. Visibility measurement of GP IOLs.

Table S1. Specifications of the laser interference lithography system.

Movie 1. Wavefronts of the transmitted Ex components for circularly polarized incident

Movie 2. Defocus images of the bifocal and trifocal GP IOLs.


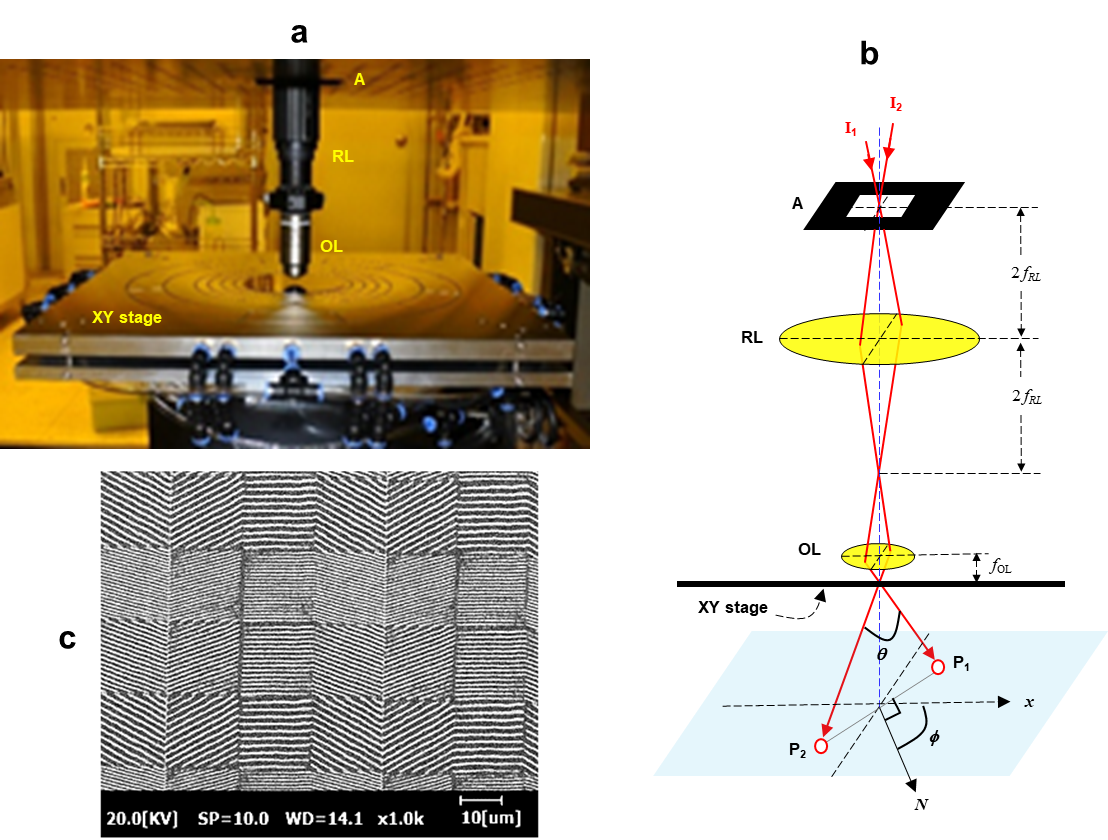


**Figure S1. Laser interference lithography system**. **a,b**, Photograph and schematic of the laser interference lithography system used for the fabrication of microtextured grating pixels. The system consists of a square aperture (A), relay lens (RL), objective lens (OL), and motorized linear stage (XY stage). The two coherent beams (I_1_ and I_2_) incident to A with 1 mm^2^ area make an interference pattern on the surface of the XY stage via RL and OL. By changing the angles of *θ* and *φ* the period (*Λ*) and orientation (*φ*) of the square gratings can be precisely defined. **c,** SEM image of a typical array of square gratings fabricated by the lithography system, where the *Λ* and *φ* are randomly arranged from 0.3 μm ~ 1 μm and 0^o^ ~ 180^o^, respectively.


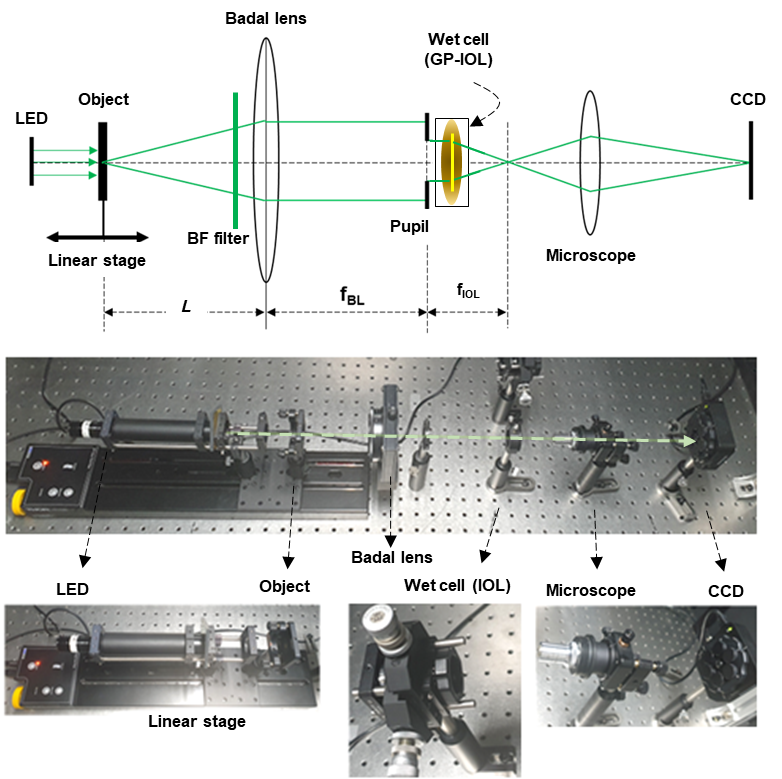


**Figure S2.** **Badal imaging system**. Schematic and photograph of the defocus imaging setup. A white LED lamp and a bandpass (BF) filter illuminates the system at 546 nm. A test object is mounted on a linear motorized translation stage (travel range 300 mm) and placed at the front focal plane of the achromatic Badal lens with 160 mm focal length (f_BL_). The GP IOL to be tested is immersed in a wet cell with saline solution with pupil sizes ranging from 2 mm to 6 mm. A 12-bit CCD beam profiler (BC106N-VIS, Thorlabs) with an image sensor having 1360 x 1024 pixels (pixel pitch of 6.45 μm); attached to a 5x microscope is used to capture the image formed by the MIOL under test. To assess the MTF, the object (a cross-hair slit with 25 μm width) plane is axially displaced to generate the different eye vergences ranging from -5 D to +5 D. The United States Air Force (USAF) 1951 Resolution Target is used in the imaging test.


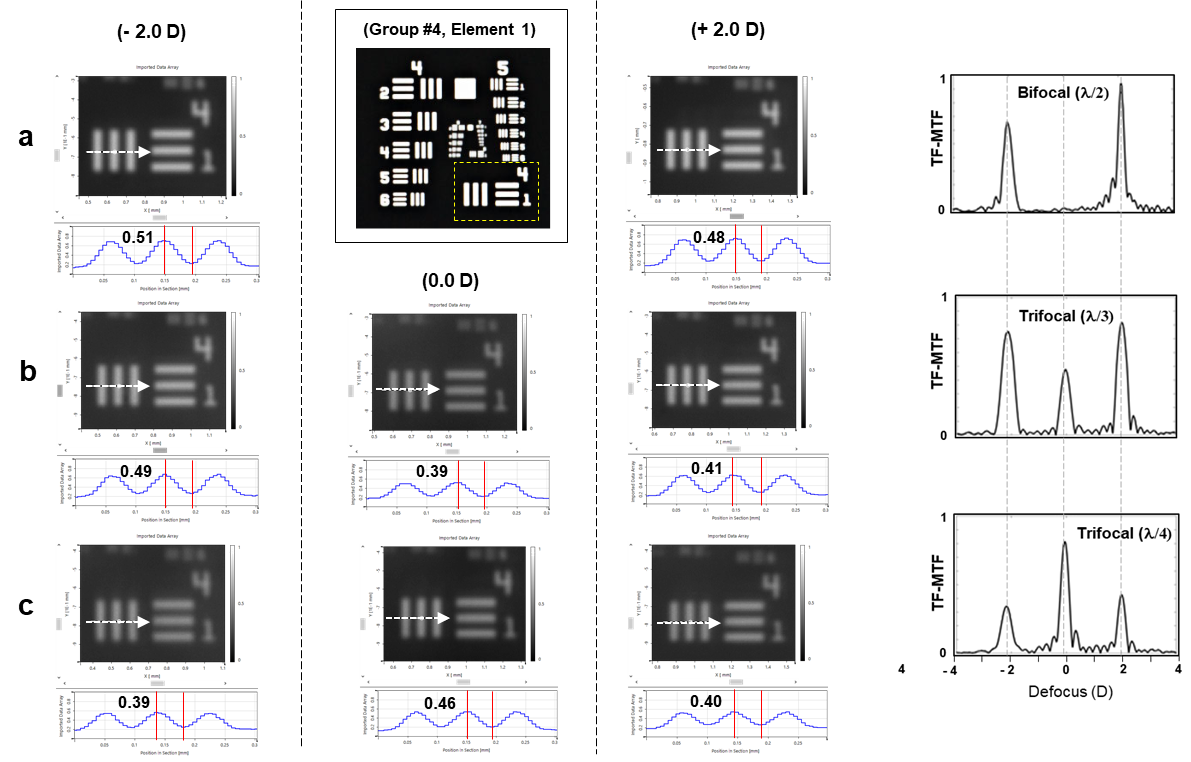


**Figure S3. Visibility measurement of GP IOLs.** Magnified images of the 16 lines mm^-1^ bar patterns (Group #4, Element 1 of the resolution target as shown in the inset) at the best defocus planes of -2.0 D, 0 D, and +2 D. **a,** bifocal (λ/2), **b,** trifocal, (λ/3), and **c,** trifocal (λ/4) GP IOLs. The line profiles are the intensity (*I*) distributions along the dashed arrows in the magnified images, where the vertical red lines indicate the positions of *I*_min_ and *I*_max_. The measured **v**isibility (contrast), defined as the ratio of (*I*_max_ - *I*_min_) (*I*_max_+ *I*_min_)^-1^, is presented in each panel of the line profile.

**Table S1. Specifications of the laser interference lithography system.**


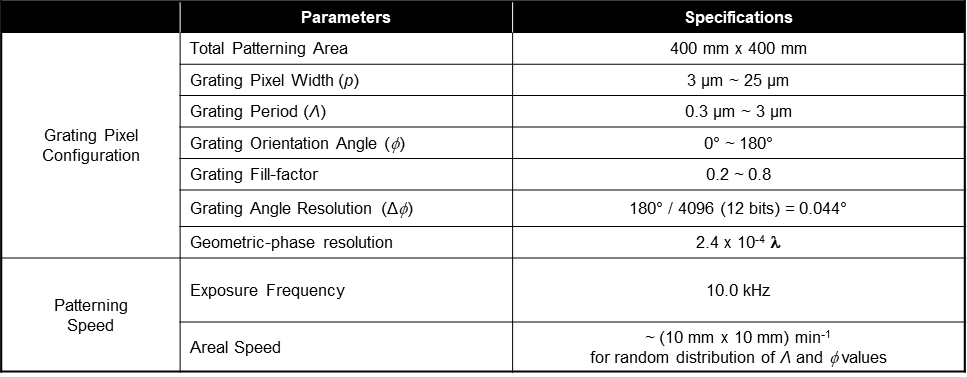


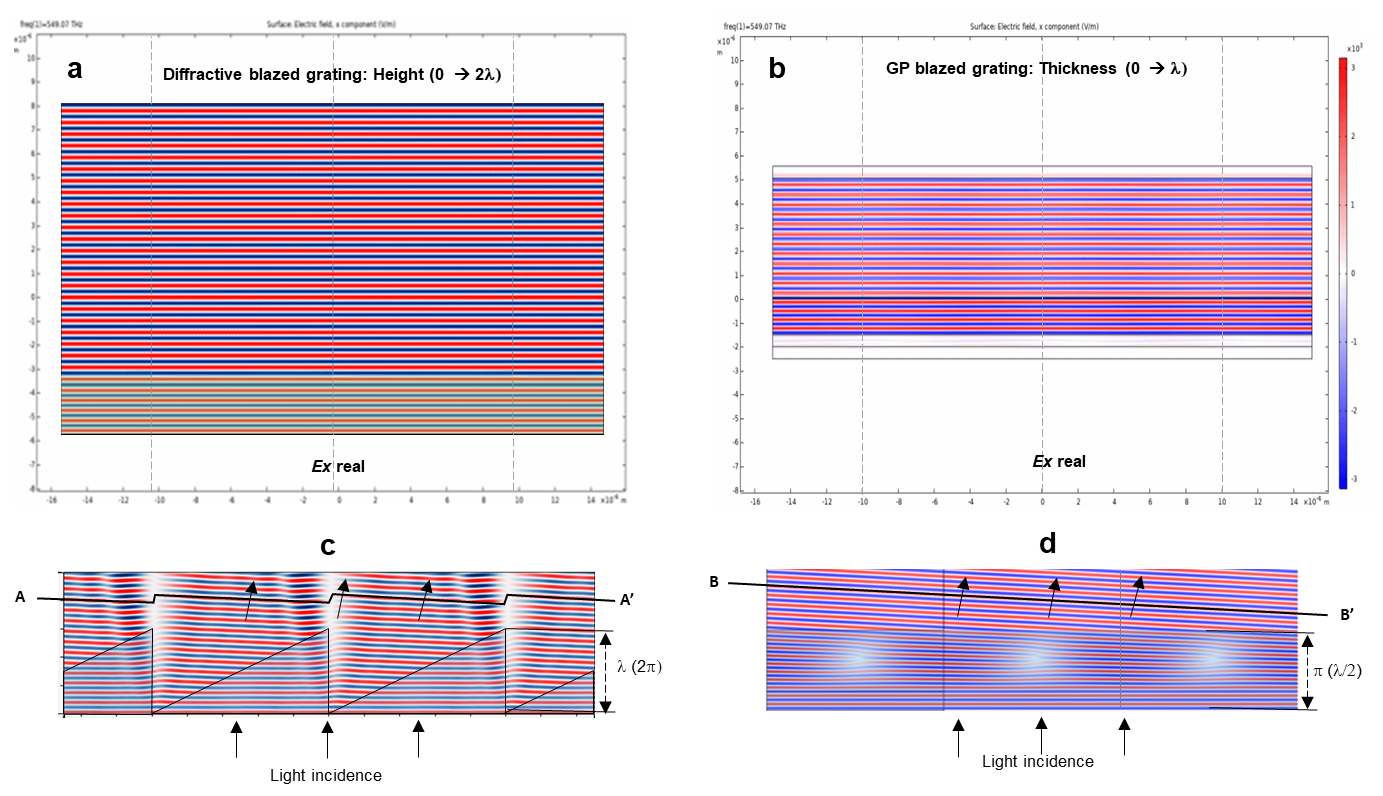


**Movie 1. Wavefronts of the transmitted Ex components for circularly polarized incident**. **a,** (Movie) Diffractive blazed grating as the height continues to increase from 0 to 2λ. **b,** (Movie) GP blazed grating as the GP phase increases from 0 to 2π. **c,** For a diffractive blazed height of λ (2π) the wavefront is stepwise along the line A-A’. **d,** For a GP blazed grating of 2π the wavefront is flat along the line B-B’.

*Please see the movie file of “Movie 1. Wavefronts of the transmitted Ex components for circularly polarized incident.mp4”.*

**
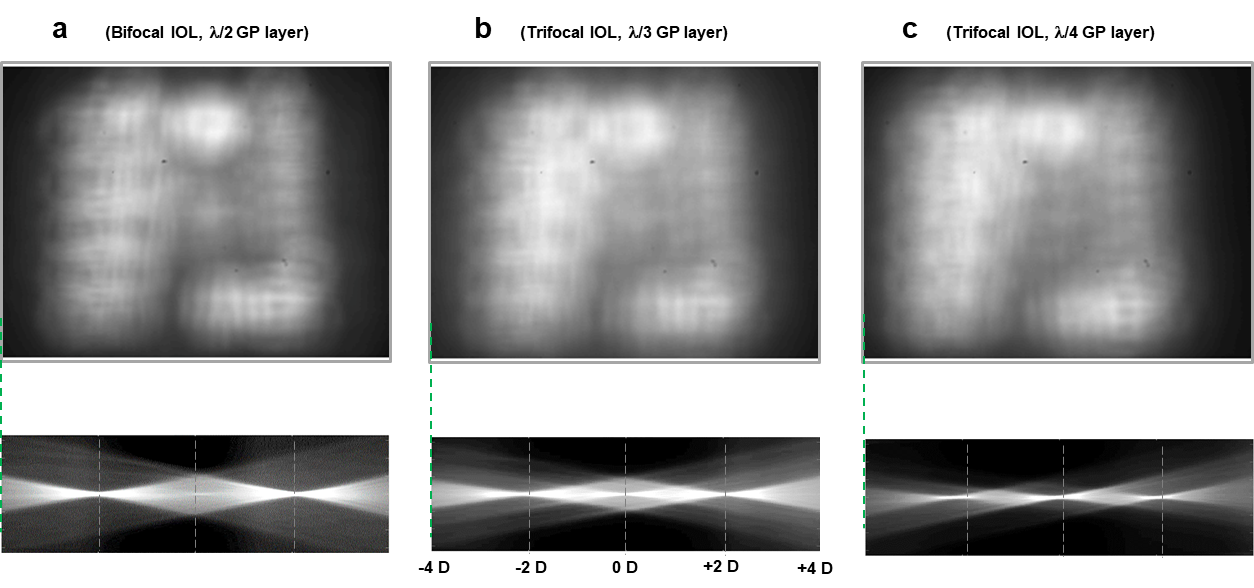
**

**Movie 2. Defocus imaging of bifocal and trifocal GP IOLs.**

*Please see the movie file of “Movie 2. Defocus imaging of the bifocal and trifocal GP IOLs.mp4”.*
